# Supplementary material for: Circulating whole genome miRNA expression corresponds to progressive right ventricle enlargement and systolic dysfunction in adults with tetralogy of Fallot
Source: PLoS One. 2020 Nov 11;15(11):e0241476. doi: 10.1371/journal.pone.0241476 (PMC7657553; doi:10.1371/journal.pone.0241476)
Supplement: S1 File — (PDF) [file pone.0241476.s001.pdf]

Supplemental Results

Supplemental Table 1. Comparison of TTE measures of right ventricular end diastolic area (RVEDA) (cm^2), RV end systolic area (RVESA) (cm^2), and RV fractional area change (RVFAC), with cardiac MRI measures of RV end diastolic volume (RVEDV) (ml), RV end systolic volume (RVESV) (ml), and RV ejection fraction (RVEF%).

	TTE			Cardiac MRI		
TTE Group	RVEDA (cm2)	RVESA (cm2)	RVFAC	RVEDV (ml)	RVESV (ml)	RVEF %
Mild/Mod	43	27	37	211	130	39
Mild/Mod	36	17	52	237	145	39
Mild/Mod	33	20	41	240	146	39
Severe	52	37	28	285	180	37

Supplemental Table 2. Top 20 Upregulated miRNA

miRNA	Corrected P – ANOVA between all groups	FC (Mild/Mod] vs [Normal])	FC (Severe] vs [Normal])	Sequence
hsa-miR-431-3p	0.0115310	34.2	78.5	AGAAGCCCTGCAAGACG
hsa-miR-196a-3p	0.0115310	29.2	72.5	CTCAGGCAGTTTCTT
hsa-miR-767-3p	0.0115310	11.3	66.6	AGAAACCATGGGGTATGA
hsa-miR-433-3p	0.0115310	11.9	39.1	ACACCGAGGAGCCC
hsa-miR-676-3p	0.0314191	12.3	36.9	AACTCAACAACCTTAGGA
hsa-miR-632	0.0263168	13.7	32.2	TCCCACAGGAAGCAGAC
hsa-miR-604	0.0160920	19.5	31.6	GTCCTGAATTCCGCAGC
hsa-miR-219a-2-3p	0.0295370	10.8	27.3	ACAGATGTCCAGCCAC
hsa-miR-520a-5p	0.0202035	9.9	25.2	AGAAAGTACTTCCCTCTGG
hsa-miR-635	0.0147121	12.4	24.3	GGACATTGTTTCAGTGCCCA
hsa-miR-541-3p	0.0267067	13.5	23.8	AGTCCAGATTCTGTGCCC
hsa-miR-371b-3p	0.0115310	5.4	21.6	GCACTCAAACTGTGGG
hsa-miR-92b-5p	0.0181049	7.2	20.8	CACTGCACCGCGTC
hsa-miR-492	0.0185653	7.7	20.7	AAGAATCTTGTCCCGCAGG
hsa-miR-381-5p	0.0160920	7.3	19.2	ATATACAAAGGGCAACCTC
hsa-miR-205-5p	0.0477631	14.5	16.2	CAGACTCCGGTGGAAT
hsa-miR-485-5p	0.0189460	4.9	13.4	GAATTCATCACGGCCAGC
hsa-miR-138-5p	0.0489596	9.5	12.9	CGGCCTGATTCACA
hsa-miR-143-5p	0.0317364	5.1	9.8	ACCAGAGATGCAGCACT
hsa-miR-28-3p	0.0003685	4.6	6.1	TCCAGGAGCTCACA

Supplemental Table 3. Top 20 Downregulated miRNA

miRNA	Corrected P – ANOVA between all groups	FC ([Mild/Mod] vs [Normal])	FC ([Severe] vs [Normal])	Sequence
hsa-miR-466	0.0116555	-151.0	-1671.6	ATGTGTGTTGCGTGTATG
hsa-miR-449b-3p	0.0115669	-127.2	-1318.3	AGTGGCAGGGTAGTTG
hsa-miR-34b-3p	0.0115310	-154.0	-1045.5	ATGGCAGTGGAGTTAGT
hsa-miR-451b	0.0115310	-105.8	-904.7	AATGGTAATGGTTCTCTTGC
hsa-miR-449c-3p	0.0140814	-82.8	-855.3	ACAGAGAGGAGTGCAAC
hsa-miR-764	0.0115310	-118.6	-810.0	AGGAGGACAAGTGAGC
hsa-miR-494-5p	0.0145486	-76.5	-533.8	AGAGAAGACAACACGGA
hsa-miR-609	0.0136966	-69.8	-458.0	AGAGATGAGAGAAACACCC
hsa-miR-647	0.0115310	-85.9	-438.9	GAAGGAAGTGAGTGCAG
hsa-miR-548aa	0.0115310	-73.8	-389.6	TGGTGCAAAAGTAATTGTG
hsa-miR-33a-3p	0.0115310	-71.9	-384.5	GTGATGCACTGTGGAA
hsa-miR-134-3p	0.0115310	-67.4	-348.4	TTGGTGACTAGGTGGC
hsa-miR-568	0.0115310	-66.7	-337.6	GTGTGTATACATTTATACAT
hsa-miR-548v	0.0115310	-63.6	-318.4	TGGTGCAAAAGTAACTGT
hsa-miR-876-5p	0.0115310	-62.4	-317.3	TGGTGATTCACAAAGAAATCCA
hsa-miR-130a-5p	0.0137388	-44.3	-247.7	GCAGACAGTAGCACAATG
hsa-miR-511-3p	0.0139931	-38.0	-203.2	TCTGTCTTTTGCTACACA
hsa-miR-885-5p	0.0489596	-64.1	-174.2	AGAGGCAGGGTAGTGTA
hsa-miR-548au-3p	0.0115669	-38.1	-174.2	CTGGTGCAAAAGTAACT
hsa-miR-943	0.0115669	-38.0	-170.1	CTGGAGGACGGCAA

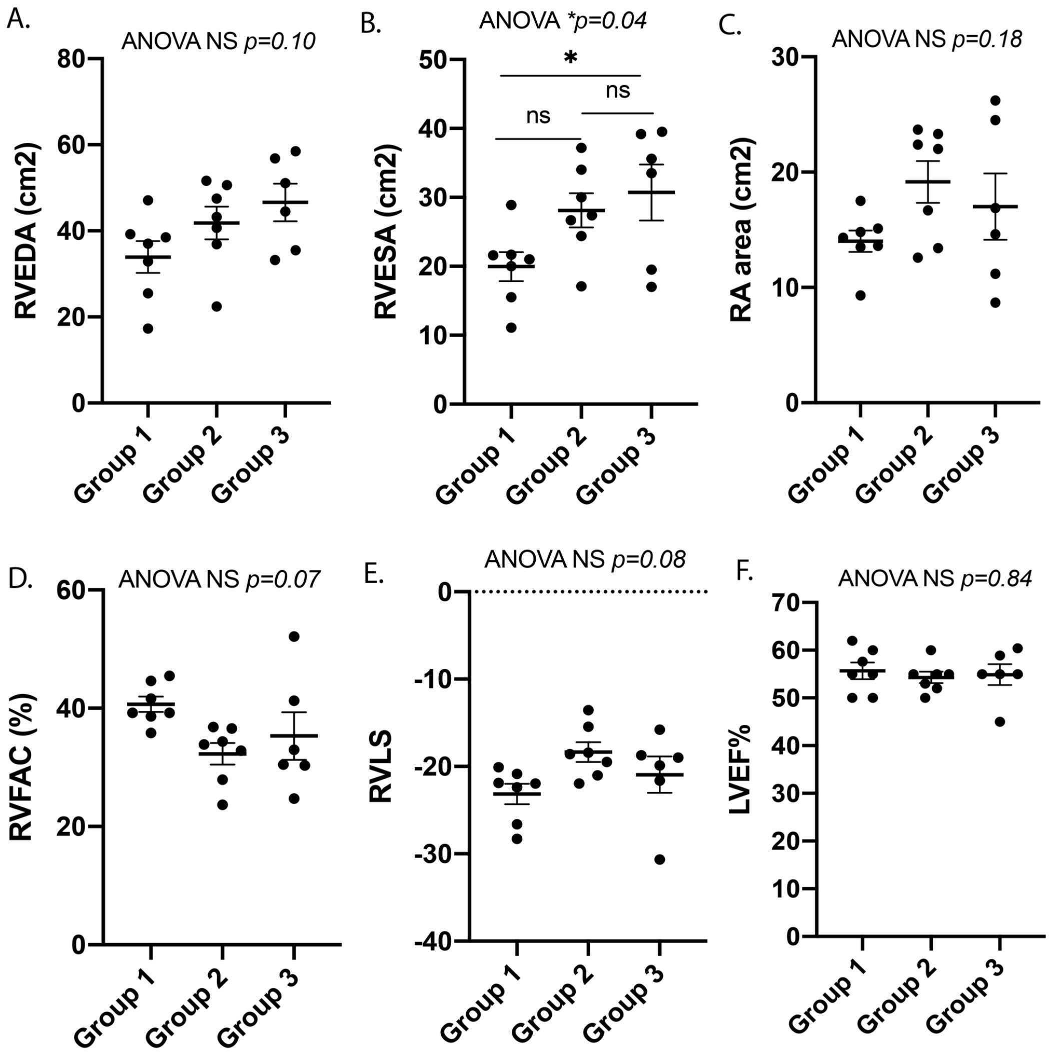

Supplemental Figure 1. Echocardiographic data based on hierarchical clustering groups show that groups correspond to RVESA. Echocardiographic characteristics based on unbiased hierarchical clustering of miRNA into 3 groups. (A) RV end diastolic area (cm^2) (RVEDA) trended toward increasing with Group 2 and 3, (B) RV end systolic area (cm^2) (RVESA) increased with Group 2 and 3, (C) right atrial area (cm^2) (RA) did not change between the groups, (D) RV fractional area change (RVFAC) trended toward decreasing with Group 2 and 3, (E) RV free wall Lagrangian longitudinal strain (RVLS) trended toward increasing with Group 2 and 3, and (F) LV ejection fraction (LVEF%) did not change between the groups. RV – right ventricle. Data is presented as mean±SEM.

Supplemental Table 4. Top 25 Dysregulated pathways from upregulated miRNA

KEGG pathway	p-value	#genes	#miRNAs
**Fatty acid biosynthesis**	4.793E-09	1	1
**Adherens junction**	8.497E-08	30	13
**Cell cycle**	1.143E-07	51	13
**Viral carcinogenesis**	1.617E-07	59	17
**ECM-receptor interaction**	1.087E-06	24	15
**Glioma**	2.079E-06	26	12
**Hepatitis B**	6.485E-06	45	13
**Proteoglycans in cancer**	6.485E-06	60	15
**Hippo signaling pathway**	1.217E-05	44	16
**Pancreatic cancer**	1.445E-05	27	11
**Pathways in cancer**	2.787E-05	100	16
**Bladder cancer**	3.052E-05	19	14
**Protein processing in endoplasmic reticulum**	4.786E-05	53	13
**Chronic myeloid leukemia**	5.405E-05	29	11
**Transcriptional misregulation in cancer**	9.525E-05	45	13
**Oocyte meiosis**	1.195E-04	36	13
**HTLV-I infection**	1.195E-04	74	17
**p53 signaling pathway**	3.962E-04	26	12
**Bacterial invasion of epithelial cells**	7.413E-04	26	13
**Shigellosis**	7.681E-04	23	11
**HIF-1 signaling pathway**	7.768E-04	36	11
**FoxO signaling pathway**	1.132E-03	42	13
**Prostate cancer**	1.856E-03	31	12
**TGF-beta signaling pathway**	3.479E-03	22	11
**Epstein-Barr virus infection**	3.479E-03	57	16

Supplemental Table 5. Top 25 Dysregulated pathways from downregulated miRNA

KEGG pathway	p-value	#genes	#miRNAs
**Cell cycle**	4.008E-11	83	34
**Lysine degradation**	5.202E-10	31	29
**Hippo signaling pathway**	5.380E-09	79	34
**Adherens junction**	1.850E-08	49	33
**Prion diseases**	9.956E-08	16	21
**Proteoglycans in cancer**	2.372E-07	100	36
**TGF-beta signaling pathway**	2.757E-07	47	31
**Chronic myeloid leukemia**	1.468E-06	49	32
**Spliceosome**	6.015E-06	77	34
**p53 signaling pathway**	6.049E-06	47	28
**Protein processing in endoplasmic reticulum**	1.359E-05	94	34
**Shigellosis**	2.990E-05	41	28
**Viral carcinogenesis**	4.580E-05	104	37
**Fatty acid biosynthesis**	4.763E-05	6	9
**ECM-receptor interaction**	6.045E-05	36	23
**Neurotrophin signaling pathway**	6.575E-05	69	33
**Bacterial invasion of epithelial cells**	6.575E-05	46	28
**RNA transport**	8.471E-05	91	37
**FoxO signaling pathway**	1.179E-04	74	32
**Pancreatic cancer**	1.603E-04	40	29
**Hepatitis B**	2.465E-04	74	35
**Glioma**	2.465E-04	37	30
**Oocyte meiosis**	3.009E-04	59	33
**Prostate cancer**	4.812E-04	53	31
**Long-term depression**	5.929E-04	29	25

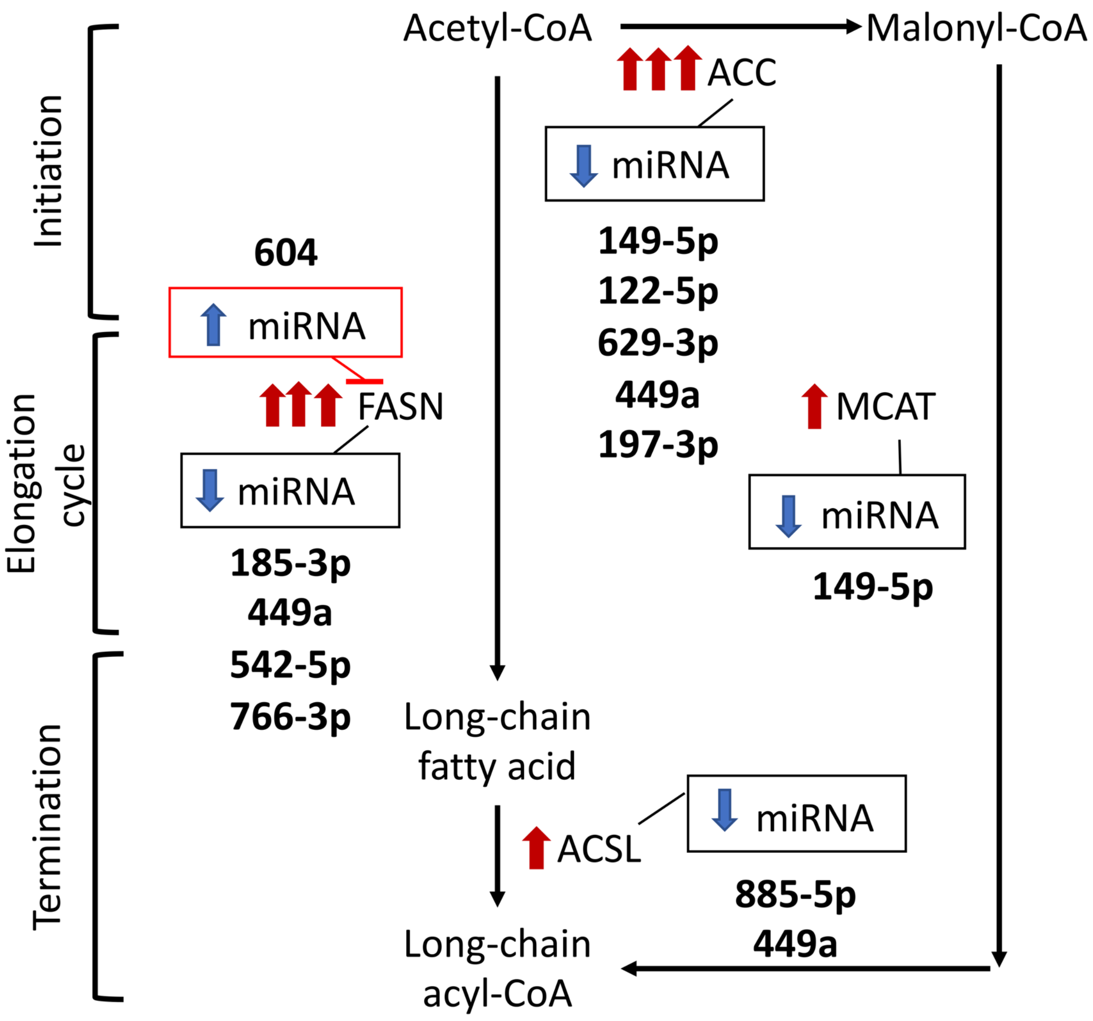

Supplemental Figure 2. Schematic of fatty acid metabolism pathway of dysregulated miRNA show net effect to enhance fatty acid metabolism. Up and downregulated miRNA target specific genes within fatty acid metabolism pathway, with net effect appearing to increase FASN, ACC, MCAT, and ACSL expression. FASN – fatty acid synthase; ACC – Acetyl-CoA Carboxylase A; MCAT – Malonyl CoA Acyl Carrier Protein Transacylase; ACSL – Acyl-CoA Synthetase Long Chain.

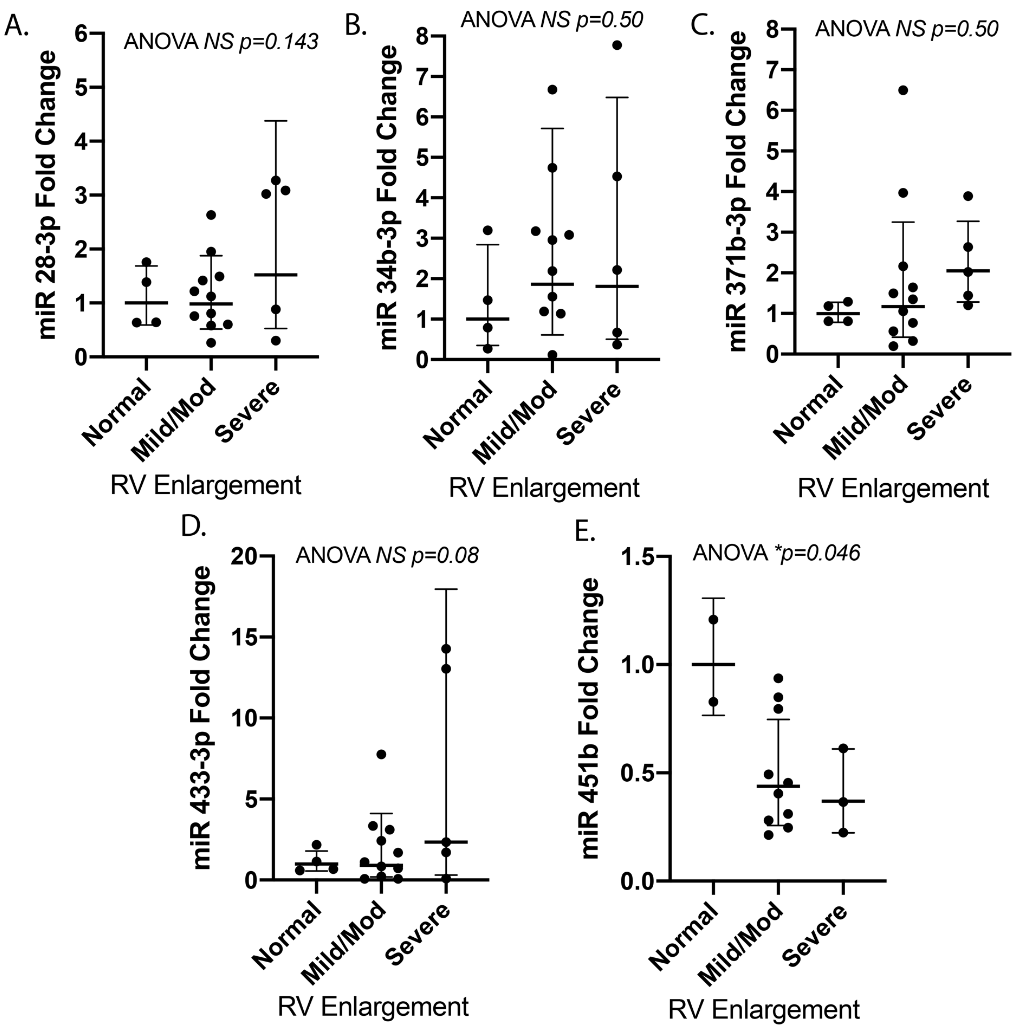

Supplemental Figure 3. qPCR validation of select miRNA show strong concordance with microarray data. (A) miR-28-3p, (B) miR 34-3p, (C) miR 371b-3p and (D) miR 433-3p trended toward increasing expression with increasing RV enlargement while, (E) miR 451b decreased with increasing RV enlargement. miR – microRNA. Data is presented as geometric mean +/- SD.

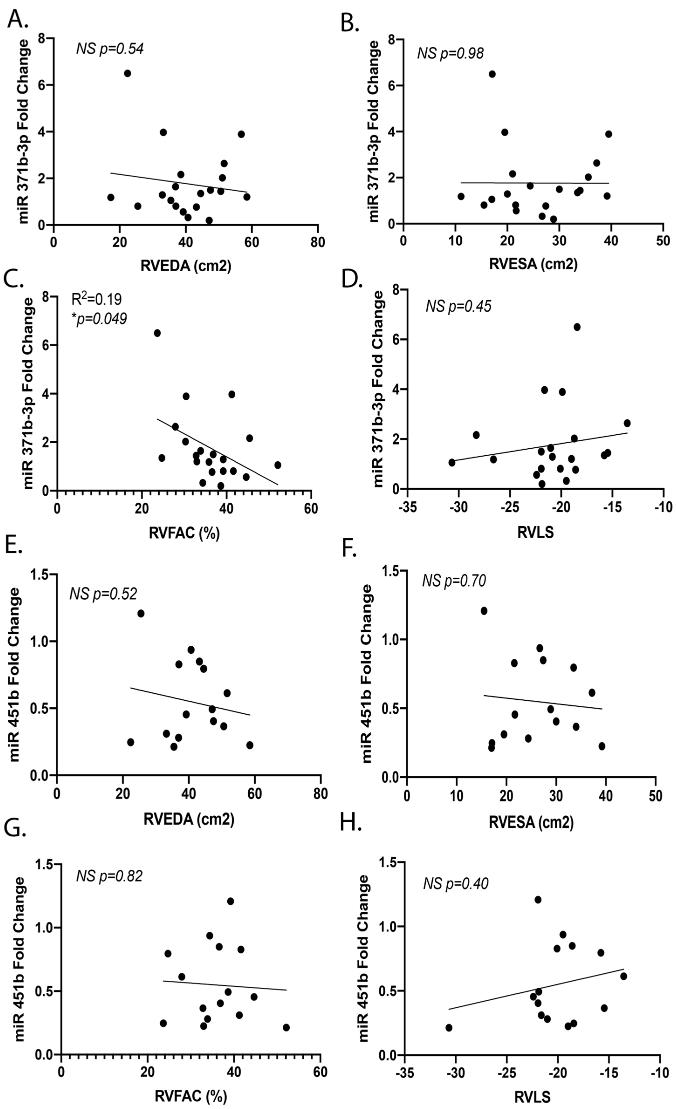

Supplemental Figure 4. miRNA 371b-3p has linear relationship to RVFAC but not RV size whereas miRNA 451b has minimal relationship. Increasing expression of miR 371b-3p does not appear to have any relationship to (A) RVEDA and (B) RVESA. (C) Increasing miR 371b-3p expression is associated with decreasing RVFAC, (D) but is not associated with RVLS. (E) Expression of miR 451b did not have significant linear relationships to RVEDA, (F) RVESA, (G) RVFAC or (H) RVLS. Data is shown as dot plot with simple linear regression (*p<0.05,**p<0.01). RV – right ventricle, RVEDA – RV end diastolic area, RVESA – RV end systolic area, RVFAC – RV fractional area change, RVLS – RV longitudinal strain.
